# Spinocerebellar ataxia type 29 due to mutations in *ITPR1*: a case series and review of this emerging congenital ataxia

**DOI:** 10.1186/s13023-017-0672-7

**Published:** 2017-06-28

**Authors:** Jessica L. Zambonin, Allison Bellomo, Hilla Ben-Pazi, David B. Everman, Lee M. Frazer, Michael T. Geraghty, Amy D. Harper, Julie R. Jones, Benjamin Kamien, Kristin Kernohan, Mary Kay Koenig, Matthew Lines, Elizabeth Emma Palmer, Randal Richardson, Reeval Segel, Mark Tarnopolsky, Jason R. Vanstone, Melissa Gibbons, Abigail Collins, Brent L. Fogel, Care4Rare Canada Consortium, Tracy Dudding-Byth, Kym M. Boycott

**Affiliations:** 1 Department of Genetics, Children’s Hospital of Eastern Ontario, Ottawa, ON Canada; 2 Greenwood Genetic Center, Greenwood, SC USA; 3 Pediatric Movement Disorders, Neuropediatric Unit, Shaare Zedek Medical Center, Jerusalem, Israel; 4 Children’s Hospital of Eastern Ontario Research Institute, Ottawa, ON Canada; 5 Carolinas Healthcare System, Charlotte, NC USA; 6 Hunter Genetics, Newcastle, NSW Australia; 7 University of Texas Health Science Center at Houston, Houston, TX USA; 8 Genetics of Learning Disability (GOLD) Service, Waratah, NSW Australia; 9 University of New South Wales, Randwick, Sydney, Australia; 10 Gillette Children’s Specialty Healthcare, St Paul, MN USA; 11 Shaare Zedek Medical Center and the Hebrew University School of Medicine, Jerusalem, Israel; 12 Department of Pediatrics, Division of Neuromuscular and Neurometabolic Diseases, McMaster University Medical Centre, Hamilton, ON Canada; 13 Department of Neurology, Children’s Hospital Colorado, University of Colorado, Denver, School of Medicine, Aurora, CO USA; 14 Departments of Pediatrics and Neurology, Children’s Hospital Colorado, University of Colorado, Denver, School of Medicine, Aurora, CO USA; 15 Program in Neurogenetics, Departments of Neurology and Human Genetics, David Geffen School of Medicine, University of California Los Angeles, Los Angeles, CA USA; 16 University of Newcastle Australia, Grow Up Well Priority Research Centre & Hunter Genetics & NSW Genetics of Learning Disability (GOLD) Service, Waratah, NSW Australia

**Keywords:** Human phenotype ontologies, Congenital non-progressive spinocerebellar ataxia, Spinocerebellar ataxia type 29, SCA29, Cerebellar atrophy, ITPR1, Clinical management

## Abstract

**Background:**

Spinocerebellar ataxia type 29 (SCA29) is an autosomal dominant, non-progressive cerebellar ataxia characterized by infantile-onset hypotonia, gross motor delay and cognitive impairment. Affected individuals exhibit cerebellar dysfunction and often have cerebellar atrophy on neuroimaging. Recently, missense mutations in *ITPR1* were determined to be responsible.

**Results:**

Clinical information on 21 individuals from 15 unrelated families with *ITPR1* mutations was retrospectively collected using standardized questionnaires, including 11 previously unreported singletons and 2 new patients from a previously reported family. We describe the genetic, clinical and neuroimaging features of these patients to further characterize the clinical features of this rare condition and assess for any genotype-phenotype correlation for this disorder. Our cohort consisted of 9 males and 12 females, with ages ranging from 28 months to 49 years. Disease course was non-progressive with infantile-onset hypotonia and delays in motor and speech development. Gait ataxia was present in all individuals and 10 (48%) were not ambulating independently between the ages of 3–12 years of age. Mild-to-moderate cognitive impairment was present in 17 individuals (85%). Cerebellar atrophy developed after initial symptom presentation in 13 individuals (72%) and was not associated with disease progression or worsening functional impairment. We identified 12 different mutations including 6 novel mutations; 10 mutations were missense (with 4 present in >1 individual), 1 a splice site mutation leading to an in-frame insertion and 1 an in-frame deletion. No specific genotype-phenotype correlations were observed within our cohort.

**Conclusions:**

Our findings document significant clinical heterogeneity between individuals with SCA29 in a large cohort of molecularly confirmed cases. Based on the retrospective observed clinical features and disease course, we provide recommendations for management. Further research into the natural history of SCA29 through prospective studies is an important next step in better understanding the condition.

**Electronic supplementary material:**

The online version of this article (doi:10.1186/s13023-017-0672-7) contains supplementary material, which is available to authorized users.

## Background

Spinocerebellar ataxias (SCA) are a clinically heterogeneous group of autosomal dominant neurogenetic disorders causing cerebellar ataxia and extra-cerebellar central nervous system manifestations which vary by specific genetic type [1]. Ataxia in SCAs is often adult-onset and gradually progressive; spinocerebellar ataxia type 29 (SCA29 [MIM 117360]) is distinguished by congenital, non-progressive ataxia associated with infantile-onset hypotonia, gross motor delay and mild cognitive impairment. Affected individuals exhibit physical signs of cerebellar dysfunction and often have cerebellar atrophy on neuroimaging [2, 3].

Recently, we used whole-exome sequencing to implicate heterozygous missense mutations in *ITPR1* (MIM 147265) (p.V1553 M and p.N602D) as the cause of SCA29 [3]. *ITPR1* encodes a ligand-gated Ca2+ channel, inositol 1,4,5-trisphosphate receptor type 1, localized to the endoplasmic reticulum (ER) membrane, and is highly expressed in Purkinje cells in the cerebellum, where it regulates ER-stored Ca^2+^release. Both missense mutations that were previously reported in SCA29 were localized to the coupling/regulatory domain of the *ITPR1* gene product, and are hypothesized to alter calcium channel function [3]. Heterozygous deletions of this gene were already known to cause SCA15, an adult-onset, slowly-progressive ataxia. This allelic condition highlights the importance of the ITPR1-pathway in cerebellar function. Additional patients with missense mutations in *ITPR1* have subsequently been reported with clinical features consistent with SCA29, often as part of large cohorts undergoing whole-exome sequencing (WES) [4–10]. Therefore, literature on the clinical features and outcomes of SCA29 is sparse and includes patients with non-progressive congenital ataxia lacking a confirmed mutation in *ITPR1* [11–18]. We set out to further refine the clinical features and natural history of this rare cause of cerebellar ataxia. We present a case series of 15 unrelated families (21 individuals) with SCA29 caused by 12 mutations in *ITPR1,* including 6 previously-unreported mutations. We describe the genetic, clinical and neuroimaging features of these patients in detail to characterize this condition and assess for any genotype-phenotype relationships in this emerging congenital ataxia. Improved understanding of SCA29 will facilitate early diagnosis and targeted intervention.

## Methods

Individuals with *ITPR1* mutation and a diagnosis of SCA29 at 10 different centers were recruited by inviting their physicians to participate. Of the 21 individuals recruited, 8 were from The United States of America, 7 from Australia, 5 from Canada and 1 from Israel. The majority of individuals (19) were of Northern European descent with 1 individual of African-American and Northern European descent and 1 individual of Sephardic and Ashkenazi Jewish descent. Informed consent was obtained from all individuals diagnosed as part of research protocols; study designs were approved by the research board at each institution in compliance with the Helsinki Declaration. A medical history questionnaire (Additional file 1: Table S1), including relevant genetic information and clinical history, was distributed to all collaborators and de-identified data was compiled for analysis. Participants in this study were identified retrospectively based on a deleterious-appearing variant in ITPR1 identified by whole exome sequencing and clinical features overlapping with SCA29 (congenital onset ataxia); as a result, recruitment bias cannot be excluded. All sequencing was performed at the individual collaborative sites in accordance with their research or clinical protocols. DNA for sequencing was selected using a number of different capture kits, but exome sequencing was performed on a HiSeq platform for all cases. Informatics pipelines used to identify variants were site-specific. Variants in SCA29 were validated by Sanger sequencing in instances where the clinical laboratory did not have an established policy for waiving Sanger sequencing. A total of 13 new cases are identified and clinical information from 2 previously reported families (8 individuals) described in Dudding and Huang et al. was also included, with updated clinical data on Family A [2, 3].

## Results and discussion

### Molecular findings

Our cohort consisted of 9 males and 12 females with heterozygous mutations identified in *ITPR1* from 15 unrelated families, including 2 families that had been previously reported [2, 3]. Of the 12 different mutations reported in our cohort, 10 were missense (4 present in >1 individual), 1 a splice site mutation leading to an in-frame insertion and 1 in-frame deletion (Table 1). Of the 10 sporadic mutations, 8 were de novo based on parental testing, with the remaining 2 mutations being classified as sporadic based on reported negative family history. We acknowledge that non-penetrance or reduced expressivity cannot be excluded in these two instances, however, both of these mutations were recurrent providing evidence for pathogenicity. Of the 12 mutations in our cohort, 10 were classified as pathogenic or likely pathogenic and all of the 13 new index individuals had pathogenic or likely pathogenic mutations (Additional file 1: Table S2).

**Table 1:** Mutations in *ITPR1* in 21 individuals with SCA29 reported in our cohort

Protein	Domain	c. DNA	Inheritance	Frequency (21)	Previously Reported
p.T267 M	IP3	c.800C > T	2 Sporadic	2	[4]
p.R269W	IP3	c.805C > T	de novo	1	[10]
p.R269G	IP3	c.805C > G	de novo	1	
p.S277I	IP3	c.830G > T	de novo	1	[4, 6]
p.K279E	IP3	c.835A > G	de novo	1	
p.K417_					
K418ins	IP3	c.1252-1G > T	de novo	1	
p.N602D	Coupling/Reg	c.1804A > G	Inherited	1	[3, 5]
p.T1386 M	Coupling/Reg	c.4157C > T	de novo	1	
p.V1553 M	Coupling/Reg	c.4657G > A	Inherited	7	[2, 3, 7]
p.G2506R	Transmembrane	c.7516G > A	1 de novo; 1 Inherited	2	
p.I2550T	Transmembrane	c.7649 T > C	2 Sporadic	2	
p.K2563del	Transmembrane	c.7687_7689del	de novo	1	[20, 21]

NM_001099952.2

**Fig. 1:**
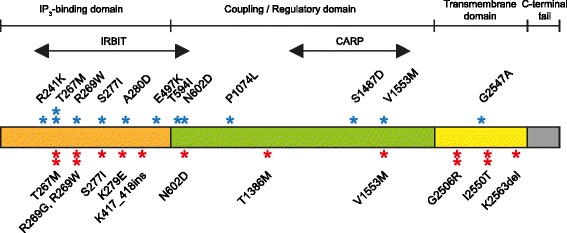
Location of mutations within all 3 functional domains of the *ITPR1* gene product (NM001099952.2). Three major domains represented including the IP_3_-binding domain (1–576), coupling/regulatory domain (576–2276), transmembrane domain (2276–2590) and C-terminal tail (2590–2749). Inhibitory binding sites IRBIT (224–604) and CARP (1387–1647) are indicated. Red asterisks represent mutations described in this study. Previously reported mutations with a SCA29 phenotype are highlighted by blue asterisks [4, 5, 7–10, 24]. Multiple asterisks at a mutation indicate recurrence in unrelated patients

The primary structure of IP3R1, the *ITPR1* gene product, consists of 3 domains including an inositol trisphosphate (IP_3_) binding domain, a coupling/regulatory region and a transmembrane spanning domain. The locations of the mutations reported in this paper and elsewhere are shown in Fig. 1. IP_3_ binding regulates the ion channel allowing for efflux of calcium ions from the ER. Additionally, IP_3_ binding is competitively inhibited at IRBIT (inositol triphosphate receptor binding protein) and CARP (carbonic anhydrase-related protein VIII) binding sites located at the opposite ends of the coupling regulatory domain [19]. Of the mutations identified in our cohort, 6 were present in the inhibitory IRBIT-binding domain, 3 in the transmembrane region and 3 were located in the inhibitory CARP-binding domain within the regulatory/coupling domain (Fig. 1). The mutations p.T267 M, p.G2506R, p.I2550T were observed twice within unrelated individuals within our cohort and 4 mutations in our cohort (p.T267 M, p.S277I, p.N602D, p.V1553 M) had previously been reported in individuals with a SCA29 phenotype [3–8]. The mutation p.K2563del was proposed to result in an in-frame deletion of a single lysine residue. This mutation has been recently reported in patients with Gillespie syndrome and was observed in our patient with aniridia [20, 21]. Interestingly, the splice site mutation c.1252-1G > T, was found to cause an in-frame 11 amino acid insertion using RT-PCR, p.K417_K418insRLSHKDHLEFH. None of the observed mutations are predicted to result in a premature stop-gain, which is consistent with the proposed mechanism of deregulation of ITPR1, and not haploinsufficiency, being causative of SCA29 [3].

### Presenting features

Individual ages at time of last assessment ranged from 28 months to 49 years. The majority of individuals had an unremarkable prenatal course and uncomplicated term delivery. Symptom recognition ranged from birth to 20 months, consistent with congenital or infantile onset, a distinct clinical characteristic of this condition (Table 2). Commonly reported symptoms leading to medical attention included gross motor delay (8/21) and hypotonia (8/21). Unfortunately, the early-onset presenting features of SCA29 are not diagnostically distinct; however, the combination of symptoms warrants early consultation with neurology and genetic services for further investigation. Additional symptoms including ataxia (4/21), poor ocular fixation (2/21) and global developmental delay (3/21) were reported. Disease course was almost uniformly non-progressive (20/21) with many individuals displaying some degree of slow improvement throughout childhood. Improvements were reported across multiple domains including attainment of developmental milestones, speech and coordination in 13 individuals; however no standardized method at different time points was used to document these improvements. Improvement in coordination and motor function with increasing age has been previously reported [11, 17, 18]. It is unclear if this improvement is due to impact of early intervention or the natural history of the condition. Notably, the older generations of Family A [2, 3] did not undergo significant targeted intervention, suggesting a natural improvement in symptoms irrespective of physical therapy.

**Table 2:** Summary findings of 21 individuals with *ITPR1* mutations and SCA29

Age (at last assessment)		
1–3 years	0/21	(0%)
3–5 years	5/21	(24%)
5–12 years	7/21	(33%)
12–18 years	4/21	(19%)
> 18 years	5/21	(24%)
Gender		
Male	9/21	(43%)
Female	12/21	(57%)
Symptoms at presentation^a^		
Hypotonia	8/21	(38%)
Motor delay	8/21	(38%)
Global developmental delay	3/21	(11%)
Ataxia	4/21	(19%)
Poor ocular fixation	2/21	(10%)
Other	12/21	(57%)
Disease course		
Non-progressive	20/21	(95%)
Progressive	1/21	(5%)
Development		
Gross Motor Delay	21/21	(100%)
Fine Motor Delay	19/19	(100%)
Language Delay	19/21	(90%)
Social/Adaptive Delay	9/19	(47%)
Cerebellar dysfunction		
Nystagmus	11/21	(52%)
Saccadic Eye Movements	7/16	(44%)
Oculomotor Apraxia	6/15	(40%)
Dysmetria	15/18	(83%)
Dysarthria	16/19	(84%)
Dysdiadochokinesis	11/16	(69%)
Intention Tremor	17/21	(81%)
Gait Ataxia	21/21	(100%)
Hypotonia	17/20	(85%)
Cognitive Impairment	17/20	(85%)
Mild	12/20	(60%)
Moderate	4/20	(20%)
Severe	0/20	(0%)
Not graded	3/20	(15%)
Neuroimaging		
Findings		
Cerebellar and/or vermis atrophy	13/18	(72%)
Other	2/18	(11%)
Normal	3/18	(17%)
Progressiveness		
Progressive	5/15	(33%)
Non-progressive	4/15	(27%)
No serial	6/15	(40%)

^a^most individuals reported more than 1 symptom at presentation

### Development

Gross motor delay was present in all 21 individuals. The majority had delayed attainment of head control and none were able to sit independently by 12 months. Most were able to stand independently between 2 and 3 years of age. All individuals were eventually able to reach these gross motor milestones but were typically delayed by 6–36 months. All individuals in the cohort had delays in achieving ambulation; 11/21 walked independently at 2–7 years of age, and the remaining 10/21 were still not ambulating independently between 3 and 12 years of age. Independent ambulation has reportedly been attainable in SCA29 between the ages of 18 months and 12 years [2–5, 7, 11]. Based on current literature, it is unclear if all individuals will achieve this milestone, and the age of independent ambulation remains variable. In our cohort, physiotherapy and occupational therapy services provided individualized modifications including gait-assist devices such as walkers and ankle foot orthoses, which resulted in independent but assisted ambulation in the older individuals.

Fine motor delay of variable severity was reported in all individuals where this information was available (19/19), ranging from 2 to 4 years below age equivalent peers. Speech delay was present in 19/21 individuals. Of those, age at first word ranged from 15 months to 3 years. All individuals older than 5 years were able to form complete sentences with a slow, scanning character. In comparison to motor and speech development, social and adaptive development was relatively spared with 9/19 individuals meeting age appropriate milestones throughout childhood. This is an important feature to highlight as it suggests that the majority of individuals with SCA29 were able to function in age equivalent social situations.

Overall, individuals with SCA29 have prominent delays in gross motor, fine motor and speech development. Early recognition of delayed milestones is critical.

Early age appropriate targeted interventions, including physiotherapy, occupational therapy and speech language pathology, and referral to developmental paediatrics is suggested.

### Cerebellar features

#### Ataxia

Ataxia was identified by clinician collaborators based on individual expertise with no standardized quantitative method required for participation in our cohort; thus, inter-rater reliability cannot be ensured. Despite this potential for variability, mild to severe gait ataxia was present in all individuals (21/21), solidifying it as a definitive feature of SCA29. Ataxia was described as slowly progressive in 1 individual, leading to significant functional impairment, but improved or remained stable in the remaining 20 individuals; however no standardized method at different time points was used to document these improvements.

All members of Family A (aged 15–49 years) maintained independent ambulation with a wide-based gait after walking between 2 and 3 years of age. The affected father of the proband from Family C has also maintained independent ambulation after age 5 years [3]. Improvement in ataxia over time has been reported in a series of patients with dominantly inherited congenital onset ataxia and cerebellar hypoplasia [13, 16, 17, 22]. Overall, ataxia in SCA29 is non-progressive or improves over time but does not completely resolve.

#### Hypotonia

Of the 20 individuals in which tone was formally assessed, early onset hypotonia was observed in 17 which ameliorated in childhood in 5/20. There was no reported decreased fetal movement on prenatal ultrasounds. Interestingly, 2 individuals presented with truncal hypotonia and limb hypertonia, with truncal hypotonia improving but peripheral hypertonia persisting (ages 22 months and 5 years). The eldest individual in the cohort continued to have unilateral hypertonia; however, the age of onset was unclear. Spasticity has been reported rarely in severely disabled patients with congenital non-progressive cerebellar ataxia [11, 23]. This suggests that early onset central hypotonia which improves with age is a common feature of SCA29. Conversely, static limb hypertonia may be present or develop in a small subset of patients.

#### Additional cerebellar signs

Additional signs of impaired coordination including nystagmus, abnormal saccades, oculomotor apraxia, dysmetria, dysarthria, dysdiadochokinesis and intention tremor were variably present in all individuals and remained stable. The majority of individuals (11/21) displayed at least 4 of these 7 features. The most commonly reported cerebellar signs included dysmetria, dysarthria and intention tremor, which were present in over 75% of individuals.

### Cognition

Cognitive impairment has previously been reported in a number of spinocerebellar ataxia syndromes including non-progressive congenital ataxia [11]. The cognitive abilities in individuals with SCA29 ranged from unaffected to mild or moderate impairment (17/20). Severe cognitive impairment was not reported in our cohort. All affected school-aged individuals had personalized education modifications and were participating at or just below age-appropriate grade level most often in mainstream educational systems. In follow-up of the 7 individuals from Family A, cognitive impairment was still recognized but degree of impairment varied. Older individuals who had not completed high-school had difficulties with reading and writing. They had never worked outside the home but were married with families. Thus, it is difficult to assess the significance of this particular mutation from a cognitive perspective.

### Neuroimaging

Results of brain magnetic resonance imaging (MRI) were available for 18 individuals with SCA29, with 13 showing evidence of cerebellar atrophy, often of the superior cerebellar hemispheres and vermis. Additionally, 2 members of Family A had evidence of pontine atrophy. Of those with cerebellar atrophy, 5 had normal imaging between the ages of 6 months and 24 months with cerebellar atrophy present on repeat imaging between the ages of 28 months to 8 years. A diagnosis of cerebellar atrophy was made on initial MRI in 8 individuals, with early identification at 12 months of age in 2 individuals. Overall, the age of onset of atrophy appears variable.

A further 3 individuals had serial scans following the diagnosis of cerebellar atrophy with 2 remaining stable and 1 showing progression between the age of 1 and 5 years. Three individuals had a normal MRI (aged 3 years, 4 years and 39 years). A single individual had findings not consistently reported in SCA29 including increased extra-axial fluid with possible cerebral atrophy; however this finding has been reported in an individual with Gillespie syndrome and a heterozygous mutation in *ITPR1* [21]. Therefore, the severity and progression of cerebellar imaging findings in our case series did not appear to closely follow the development of clinical ataxia in all cases. Nevertheless, while the neuroimaging findings are not specific to SCA29, progressive cerebellar atrophy during childhood with a non-progressive clinical course was evident in our cohort.

### Other features

A number of other clinical features were present in our cohort but no particular feature occurred with enough frequency to be considered characteristic of SCA29 (Table 3). Various ophthalmologic findings were present in multiple patients including aniridia in one patient which is suggestive of Gillespie syndrome and therefore we would recommend early ophthalmologic consultation. Seizures have been reported in individuals with a clinical diagnosis of non-progressive congenital ataxia (10 individuals within a cohort of 34 patients without a molecular diagnosis) [11]. Only 2 of our cohort were reported to have seizures, 1 of whom also carried an inherited *GRIN2A* mutation which is thought to be the primary cause of her epilepsy.

**Table 3:** Frequency of additional clinical features in individuals with SCA29

Clinical Feature	Frequency reported (percent)
Eyes	
Strabismus	5/21 (24%)
Fixed mydriasis	1/21 (5%)
Aniridia	1/21 (5%)
Ptosis	1/21 (5%)
Cortical visual impairment	1/21 (5%)
ENT	
Recurrent otitis media	1/21 (5%)
Ankyloglossia	1/21 (5%)
Cardiac/Lung	
Pulmonic stenosis	1/21 (5%)
Abdominal	
Abdominal wall hernia	1/21 (5%)
GERD	3/21 (14%)
Neurologic	
Seizures^a^	2/21 (10%)
Sleep issues	2/21 (10%)
Stereotypies	1/21 (5%)
Autism spectrum disorder	1/21 (5%)
Post-natal microcephaly	1/21 (5%)
Others	
Cutaneous hemangioma	1/21 (5%)
Failure to thrive	2/21 (10%)
Urinary and fecal incontinence	1/21 (5%)

^a^Includes 1 patient with known GRIN2A mutation

### Genotype-phenotype correlation

The small number of reported patients with SCA29 makes it difficult to draw definitive conclusions regarding the clinical spectrum of this disorder. We evaluated our clinical data and were unable to find any genotype-phenotype specific correlations within SCA29. We report mutations spanning the entire gene (with the exception of the C terminal tail and the non-IRBIT section of the IP3 binding domain) with no difference noted between the clinical features arising from mutations in the various protein domains. Unrelated individuals who harboured the same mutation expressed variable signs of cerebellar dysfunction on examination, as did the members of Family A. A varying degree of cognitive impairment was also noted in the members of Family A. Interestingly, sparing of cognition has been reported in a 4 generation Russian Family harbouring the same p.V1553 M mutation as Family A [7].

### Allelic disorders

The earlier age of onset, delay in development and cognitive impairment clearly distinguish SCA29 from slowly-progressive, adult-onset SCA15. Although allelic disorders, the SCA29 *ITPR1* mutations remain in-frame and are predicted to alter ITPR1’s regulation whereas SCA15 deletions are consistent with haploinsufficiency. Two different missense variants have been reported to be associated with SCA15, however their pathogenicity is unclear. Further investigation of the p.P1074L variant with functional studies did not support pathogenicity and the second reported missense variant is difficult to interpret as clinical details were lacking beyond the phenotype “ataxia” [24–26].

Gillespie syndrome is a condition characterized by clinical features of SCA29 with an additional feature of bilateral aniridia or iris hypoplasia. A small number of specific heterozygous missense mutations, compound heterozygous and homozygous mutations in *ITPR1* have recently been implicated to cause Gillespie syndrome. These mutations are thought to cause either a complete loss of ITPR1 activity or a structure-specific disruption of protein function [20, 21]. Patient 20 from our cohort presented with features of SCA29 and bilateral aniridia. At the time of their enrollment in our study, there was no known association between *ITPR1* and Gillespie syndrome, however, their particular mutation (p.K2563del) has recently been implicated and thus their diagnosis is more in keeping with Gillespie syndrome [20, 21].

A de novo *ITPR1* missense mutation has very been implicated as causative in a single patient with severe pontine and cerebellar hypoplasia mimicking a diagnosis of pontocerebellar hypoplasia (PCH). The presentation for this patient was also nonprogressive but was much more severe in both clinical and radiological features than the patients reported here [27].

### Suggestions for diagnosis and management

Given the wide differential diagnosis for congenital or early-onset ataxia, we recommend exome sequencing or a comprehensive panel approach to increase the likelihood of a definitive diagnosis [28]. Based on our clinical data, we suggest the following guidelines for the care of individuals with SCA29:

1. Early identification of developmental delays with referral to a developmental pediatric specialist. This would include early referral to physiotherapy, occupational therapy and speech language therapy services for targeted interventions.
2. Neurologic assessment and annual follow-up for cerebellar manifestations and monitoring of the clinical course. Based on our findings, cerebellar atrophy does not correlate with impairment or alter management and therefore we defer the decision regarding the need for serial neuroimaging to the treating neurologist.
3. Ophthalmology assessment to rule out bilateral aniridia or iris hypoplasia which are indicative of a diagnosis of Gillepsie syndrome.
4. Given the high frequency of cognitive impairment in our cohort, and the potential benefits of early recognition and interventions, developmental age appropriate psychoeducational assessments of cognitive function and early-modified education supports if necessary.
5. Genetic counselling for families regarding low recurrence risk in sporadic de novo cases, and 50% in those cases in which a parent is also affected with SCA29.

## Conclusions

We have assembled a cohort of individuals with SCA29; documenting clinical features and pathogenic mutation in *ITPR1* in 21 affected individuals from 15 unrelated families. *ITPR1* mutations which remain in-frame and are predicted to alter ITPR1’s regulation are associated with an SCA29 phenotype; allelic disorders include SCA15 and Gillepsie syndrome. Existing clinical data suggest that the disease course in SCA29 is non-progressive, with infantile-onset hypotonia and delay in attaining gross, fine and speech development milestones. Gait ataxia and variable cerebellar manifestations are present in all individuals, with many eventually clattaining independent ambulation. Mild-to-moderate cognitive impairment may be present, but given appropriate developmental supports, most individuals participate at or just below age-equivalent level in school. Early and targeted intervention should be the standard of care for individuals with SCA29 due to the significant and timely gains that can be made. Based on our small cohort, cerebellar atrophy on neuroimaging appears to be detected later than clinical features are recognized, and appears not to parallel clinical symptoms or functional impairment. The most significant barrier to attaining functional independence was the presence and severity of cognitive impairment. In general, individuals with SCA29 are healthy with a good quality of life despite some impairment. Further research into the natural history of SCA29 through prospective studies is an important next step in better understanding the condition.

## Additional file

Additional file 1:
**Table S1.** SCA29 Questionnaire. **Table S2.** Assessment of pathogenicity for ITPR1 variants identified in this study. (DOCX 1606 kb)

## Abbreviations

CARP: Carbonic anhydrase-related protein VIII
ER: Endoplasmic reticulum
IP_3_: Inositol trisphosphate binding domain
IRBIT: Inositol triphosphate receptor binding protein
ITPR1: Inositol 1,4,5-trisphosphate receptor type 1
MRI: Magnetic resonance imaging
SCA: Spinocerebellar ataxia
SCA29: Spinocerebellar ataxia type 29
