# Supplementary material for: Spinocerebellar ataxia type 29 due to mutations in ITPR1: a case series and review of this emerging congenital ataxia
Source: Orphanet J Rare Dis. 2017 Jun 28;12:121. doi: 10.1186/s13023-017-0672-7 (PMC5490223; doi:10.1186/s13023-017-0672-7)
Supplement: Additional file 1: — Table S1. SCA29 Questionnaire. Table S2. Assessment of pathogenicity for ITPR1 variants identified in this study. (DOCX 1606 kb) [file 13023_2017_672_MOESM1_ESM.docx]

**Table S1:** SCA29 Questionnaire


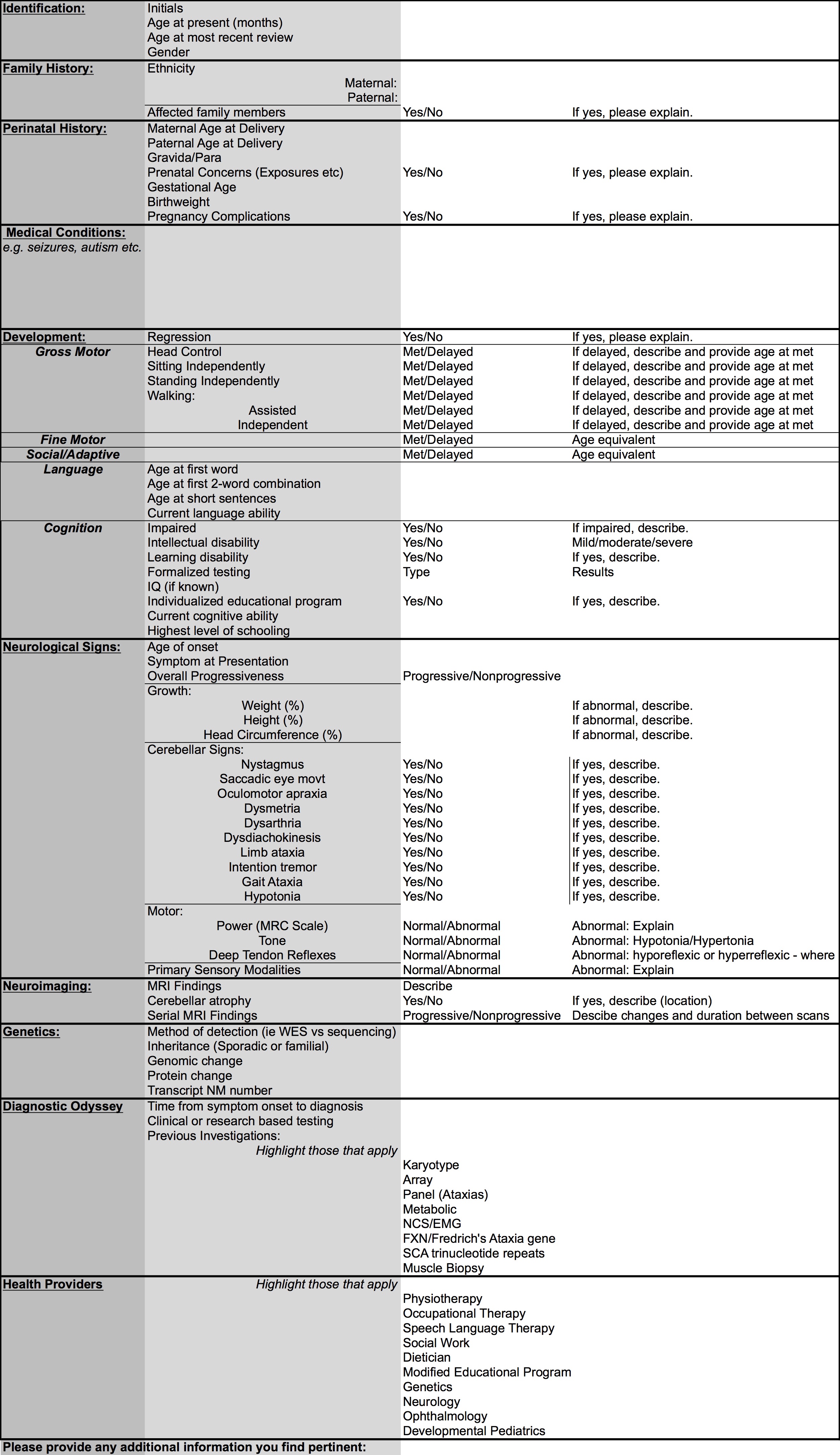


| **Variant** | **Inheritance** | **ExAC Frequency** | **In Silico Prediction*** | **Conservation** | **ClinVar** | **References** | **ACMG Classification^#^** |  |
| --- | --- | --- | --- | --- | --- | --- | --- | --- |
| p.T267M | singleton; singleton | 0 | Deleterious | Highly Conserved | Ambry Genetics; Likely Pathogenic (no date provided) | [4] | Likely Pathogenic |  |
| p.R269W | de novo | 0 | Deleterious | Highly Conserved | GeneDx; Pathogenic (June 24, 2016) | [10] | Likely Pathogenic |  |
| p.R269G | de novo | 0 | Deleterious | Highly Conserved | - |  | Likely Pathogenic |  |
| p.S277I | de novo | 0 | Deleterious | Highly Conserved | UCLA Clinical Genomics Center;  Likely Pathogenic (October 1, 2013) | [4,6] | Likely Pathogenic |  |
| p.K279E | de novo | 0 | Deleterious | Highly Conserved | - |  | Likely Pathogenic |  |
| p.K417_K418ins | de novo | 0 | Splice Impacting | Highly Conserved | - | Functional studies -aberrant transcript | Pathogenic |  |
| p.N602D | Inherited | 0 | Deleterious | Highly Conserved | - | [3,5] | VUS |  |
| p.T1386M | de novo | 0 | Deleterious | Highly Conserved | - |  | Likely Pathogenic |  |
| p.V1553M | Inherited | 0 | Deleterious | Highly Conserved | - | [2,3,7] | VUS |  |
| p.G2506R | de novo;  inherited (mat. mosaicism) | 8.28E-06 | Deleterious | Highly Conserved | - |  | Likely Pathogenic |  |
| p.I2550T | singleton; singleton | 0 | Deleterious | Highly Conserved |  |  | Likely Pathogenic |  |
| p.K2563del | de novo | 0 | Deleterious | NA | - | [20,21] | Likely Pathogenic |  |

**Table S2:** Assessment of pathogenicity for ITPR1 variants identified in this study

#Variants were classified as per ACMG guidelines [1] using the mutation analysis program Alamut Visual (V2.8.0).

*For pathogenicity prediction scores, SIFT 1.0 [2], PolyPhen 2.0 [3], and Mutation Taster [4] were considered in the calculation and consensus of all programs considered a predicted effect on protein function.

**Supplementary References**

1. Richards S, Aziz N, Bale S, Bick D, Das S, Gastier-Foster J, et al. Standards and guidelines for the interpretation of sequence variants: a joint consensus recommendation of the American College of Medical Genetics and Genomics and the Association for Molecular Pathology. Genet. Med. Springer Nature. 2015:405–24.

2. Kumar P, Henikoff S, Ng PC. Predicting the effects of coding non-synonymous variants on protein function using the SIFT algorithm. Nat Protoc. 2009;4:1073–81.

3. Adzhubei IA, Schmidt S, Peshkin L, Ramensky VE, Gerasimova A, Bork P, et al. A method and server for predicting damaging missense mutations. Nat. Methods. 2010;7:248–9.

4. Schwarz JM, Rödelsperger C, Schuelke M, Seelow D. MutationTaster evaluates disease-causing potential of sequence alterations. Nat. Methods. 2010;7:575–6.
